# P01-032 – Characterization of genetic-negative FMF

**DOI:** 10.1186/1546-0096-11-S1-A36

**Published:** 2013-11-08

**Authors:** I Ben-Zvi, C Herskovizh, Y Kassel, A Livneh

**Affiliations:** 1 Heller Institute of Medical Research, Departemnt of Medicine F, Sheba medical center, Ramat-Gan, Tel Aviv, Israel; 2 Sackler Faculty of Medicine, Tel Aviv University, Tel Aviv, Israel; 3 Departemnt of Medicine F, Sheba medical center, Ramat-Gan, Israel

## Introduction

Up to 20 percent of FMF cohorts consist of patients fulfilling the diagnostic criteria of familial Mediterranean fever, yet carry no MEFV mutations (genetic-negative). The phenotype of these patients has been poorly characterized.

## Objectives

To define clinical and demographic parameters of genetic negative FMF.

## Methods

In this observational comparative study, 47 sequential genetic negative FMF patients and 78 sequential genetic positive (for at least one allele) FMF control patients were compared using a comprehensive questionnaire completed at the time of their routine clinic visit, using direct questioning and patients' files. The definition of FMF was based on our clinical tool, widely accepted for FMF diagnosis. Absence of the 5 most common MEFV mutations in routine genetic testing of FMF was considered genetic negative FMF. Disease severity was determined by Mor criteria.

## Results

The mutation-negative and mutation positive cohorts differed respectively on the age of disease onset (19.6 vs. 10.1 years, p<0.001), family history of FMF (44% vs. 76.9%, p<0.001), rate of severe disease (23.4% vs. 64.1%, p<0.001), and rate of erysipelas-like erythema which was higher in the control group (p=0.024). There was a trend for diagnosis delay (9.95 years vs. 6.68 (p=0.08). There were no significant differences in gender and in a wide array of clinical manifestations. The average dose of colchicine, the response to treatment and the rate of chronic manifestation of FMF were also comparable between the two patient groups.

## Conclusion

The FMF specific phenotype manifested in mutation-negative FMF, together with low prevalence of family history, suggest the occurrence of a de-novo genetic event downstream the MEFV related pathway.

## Disclosure of interest

None declared.

